# Examining Working Memory Training for Healthy Adults—A Second-Order Meta-Analysis

**DOI:** 10.3390/jintelligence12110114

**Published:** 2024-11-12

**Authors:** Maria Syed, Jarrad A. G. Lum, Linda K. Byrne, David Skvarc

**Affiliations:** 1 Faculty of Health, School of Psychology, Deakin University, Melbourne 3125, Australia; 2 Faculty of Psychology, Counselling and Psychotherapy, Cairnmillar Institute, Melbourne 3123, Australia

**Keywords:** working memory training, randomized controlled trials, meta-analysis, physical activity training, mindfulness training, video game training

## Abstract

Background: Enhancing working memory performance in cognitively and physically healthy individuals is a popular area of research. The results from a large number of studies have now been summarized in multiple meta-analyses. In these reviews, various training methods have been examined, including mindfulness training, adaptive working memory training, physical activity training, and video game training, to examine whether working memory capacity can be improved. This report aggregated the results of these meta-analyses using second-order meta-analytic approaches to ascertain the extent to which working memory functioning can be enhanced in healthy adults. Methods: A total of six meta-analyses of randomized controlled trials that compared working memory interventions to a control group were included in the analyses. These studies were identified after systematically searching three electronic databases: APA PsycInfo, ERIC and Medline. Collectively, the meta-analyses investigated the effects of cognitive programs, mindfulness, video games and physical activity on working memory. Only meta-analyses undertaken with healthy adults aged between 18 and 55 years were included in the report. Results: The results revealed an average improvement in working memory across the included studies compared to the control groups. The findings indicated a small yet significant enhancement in working memory, with a standardized mean difference of 0.335 (95% CI [0.223; 0.447], *p* < .001). Further analysis tests for superiority effects between the different working memory training programs revealed no significant differences between intervention effect sizes. Conclusion: Based on the findings, it can be concluded that the working memory capacity of healthy adults can be improved through training. However, the effect size is small, so the utility of this type of training in real-life improvements in cognition may be minimal. The evidence does not indicate that one type of working memory training is superior to another.

## 1. Introduction

Working memory (WM) is a cognitive system with a limited capacity that enables individuals to hold, maintain, and manipulate goal-relevant information crucial for organizing goal-directed behaviors (Chatham and Badre 2015). WM is vital for everyday functioning, and any compromise is associated with academic difficulties, such as reading comprehension and mathematics (Gathercole and Alloway 2008). WM is linked to an individual’s fluid intelligence (Unsworth et al. 2014) and cognitive control ability (Redick and Engle 2011). The literature establishes that older participants perform poorly on WM tasks compared to younger participants, demonstrating a decline in performance with age (Ziaei et al. 2017). WM is essential for everyday activities, and extensive research has been conducted on training and improving WM capacity (Foster et al. 2017; Chein and Morrison 2010; Westerberg and Klingberg 2007; Jaeggi et al. 2008). Enhancing an individual’s WM performance can improve other cognitive abilities (Dahlin 2011; Jaeggi et al. 2008; Beck et al. 2010; Grunewaldt et al. 2013). Various training methodologies have been examined in the cognitive training literature to identify their impact on improving WM functions, such as computerized working memory training, mindfulness training, physical activity training, and video game training.

### 1.1. Adaptive Cognitive Training on WM Tasks

Previous studies have shown that participants who underwent adaptive cognitive training on WM tasks exhibited improved cognitive control and reading comprehension abilities (Chein and Morrison 2010) and were better able to process new information (Jaeggi et al. 2008). The working memory training paradigm involves the repeated practice of working memory tasks; most commonly used are the n-back and complex span tasks. Complex span tasks, for instance, train participants to temporarily hold and maintain items such as letters or words in active memory for a later recall test while simultaneously engaging in a short, distracting activity (Baddeley 1986). The N-back task is a WM training task defined as a continuous recall task that presents stimulus sequences, like letters or pictures; participants indicate whether the currently presented stimulus matches the one presented ‘n’ times ago. The training tasks are designed to be adaptive, meaning the difficulty level adjusts based on the participant’s performance. This feature ensures that the training remains cognitively demanding, constantly challenging the trainee (Klingberg 2010). It also encourages using executive functions required for the task rather than relying on a specific strategy. The consistent mismatch between the task demands and the trainee’s ability is hypothesized to lead to improvements in WM (Lövdén et al. 2010). Repeated practice with such tasks is assumed to influence WM capacity and general cognitive abilities (Unsworth et al. 2014; Redick and Engle 2011; Dahlin 2011).

However, previous studies show that repeated task practice only improves performance on the trained task, and the transfer of learning to other cognitive tasks is absent or limited (Soveri et al. 2017; Melby-Lervåg and Hulme 2013; Watrin et al. 2022). Soveri et al. (2017), in their meta-analysis of 33 published randomized controlled trials, found that WM training with n-back tasks showed a medium-sized transfer effect to untrained n-back tasks. In contrast, transfer to other cognitive abilities, such as fluid intelligence and cognitive control, was minimal. In their analysis, Melby-Lervåg and Hulme (2013) found that repeated practice of WM tasks only produces reliable short-term improvements that are not sustained at follow-up and that the transfer of training to untrained tasks is absent. Moreover, a recent two-year longitudinal study investigated the effects of WM training for near-transfer effects on WM performance and far-transfer effects on crystallized and fluid intelligence and found substantial training effects only for trained WM tasks (Watrin et al. 2022). This means that while there is some evidence of a near-transfer effect, which is enhanced performance on tasks similar to the trained task, a far-transfer effect, which is a transfer effect on tasks different from the trained task, is minimal. However, some argue that when a near-transfer effect is present, improving working memory capacity following training may be adequate to bring about meaningful differences in individuals’ daily functioning. This claim is supported by studies demonstrating improvements in mood and attention in daily life after successful WM training (Spencer-Smith and Klingberg 2015; Xiu et al. 2018; Whitelock et al. 2018; Barkus 2020; Beloe and Derakshan 2020).

### 1.2. Mindfulness Training

Various training methodologies have focused on improving working memory, including mindfulness training. Mindfulness is increasingly recognized as a valuable intervention for various purposes, given its cost-friendly and time-efficient nature (Müller et al. 2021). Derived from the Eastern Buddhist tradition, mindfulness focuses purposefully on the present moment without judgment (Kabat-Zin 2013). For example, in focused attention mindfulness meditation practice, practitioners purposefully focus on the sensations of their breathing and gently guide them back to their breathing when they become aware that their mind has wandered. Another form of mindfulness training, open monitoring, involves non-judgmentally and non-reactively observing one’s thoughts as they come and go. Mindfulness practices aim to enhance attentional control skills, increase awareness of cognitive states and emotions, and promote acceptance and non-judgmental, non-reactive awareness of the present moment (Jha et al. 2019; Lutz et al. 2008). Mindfulness training has been associated with various health benefits, including reduced symptoms of depression and anxiety (Goyal et al. 2014), improved pain management (Veehof et al. 2011), enhanced sleep quality (Black et al. 2015), decreased burnout (MacKenzie et al. 2006), increased resilience to stress (Galante et al. 2018; Shapiro et al. 2005), less distractive behaviors (Jain et al. 2007), and improved cognitive functions (Cásedas et al. 2020).

Since 2016, at least ten meta-analytic studies have examined the effects of mindfulness-based interventions on WM performance in healthy adults, but the evidence is generally inconclusive. Yakobi et al. (2021), in their meta-analysis of 27 randomized controlled trials examining the efficacy of mindfulness training in healthy adults, found limited improvements in attention and executive functions but no improvement in WM. Likewise, Cásedas et al. (2020), in a meta-analysis of randomized controlled trials examining the efficacy of mindfulness training on adults (including clinical populations), reported a small effect size for WM. Systematic reviews have also found limited improvement in WM capacity following mindfulness training and cautioned that findings may not be conclusive due to methodological limitations in individual studies (Lao et al. 2016; Chiesa et al. 2011). This lack of consistent findings has been linked to differences in methodological approaches, including variations in mindfulness programs tailored to diverse patient groups (Lao et al. 2016). Furthermore, variations in the conceptualizations and operational definitions of mindfulness have resulted in researchers developing mindfulness programs that fail to meet clinical standards (Im et al. 2021).

### 1.3. Physical Activity Training

The effects of physical activity training on various domains of cognition, including WM, have also been evaluated. Since 2016, dozens of meta-analyses studies have examined the effects of physical activity training on cognition. For example, studies over the last decade have found that moderate aerobic and resistance exercise levels can improve cognitive functions in older adults (Northey et al. 2018) and adolescents (Esteban-Cornejo et al. 2015). These benefits are associated with structural changes in the brain following physical exercise training, such as a study that found that aerobic exercise training reverses hippocampal volume loss in late adulthood, improving memory functions (Erickson et al. 2011). Regular physical activity positively influences WM performance more than acute physical activity (Rathore and Lom 2017; Haverkamp et al. 2020). A recent meta-analysis (Xue et al. 2019) found that long-term exercise improves overall executive functions, including WM, cognitive flexibility and inhibitory control.

Moreover, a meta-analysis (Rathore and Lom 2017) of fifteen randomized controlled trials compared the effects of acute (e.g., a single physical activity session) and chronic physical activity training (e.g., more than one physical activity session) on WM in physically and cognitively healthy individuals. In their study (Rathore and Lom 2017), physical activity encompassed a wide range of interventions, including traditional forms such as cardiovascular exercise and resistance training, as well as non-traditional forms such as yoga. They found a significant, small effect for chronic intervention and a non-significant effect for acute intervention. Chronic physical activity interventions lasting 4–12 weeks demonstrated stronger cognitive benefits as training effects can accumulate over time, compared to acute physical activity, which may not yield immediate benefits on working memory after just one session (Rathore and Lom 2017). Furthermore, a systematic review and meta-analysis (Alvarez-Bueno et al. 2017) found that school-based physical activity interventions can effectively enhance the cognitive development of young people. Structured physical activity interventions in schools, such as curricular physical education, active breaks, integrating physical activity into subjects like mathematics, and promoting active recess or lunchtime physical activity, have been found to have a positive impact on the working memory of children and adolescents (Alvarez-Bueno et al. 2017).

Long-term physical activity, such as chronic physical activity interventions, has been found to have a positive effect on working memory (Haverkamp et al. 2020). It creates lasting changes in the brain through neurogenesis (the development of new neurons) and synaptic plasticity (improving connections between neurons) (Hillman et al. 2015; Gomez-Pinilla and Hillman 2013). These processes improve working memory functions over time (Chang et al. 2013). Conversely, acute physical activity interventions, such as training sessions lasting less than 60 min, have been found to positively influence attention and cognitive flexibility, but not working memory (Haverkamp et al. 2020). Finally, a meta-analysis (Cai et al. 2021) of 28 randomized controlled trials examining the impact of physical activity interventions on the working memory of older adults (aged 62 to 86) concluded that regular physical activity, specifically exercising three times a week for 45–60 min at a moderate intensity, incorporating multicomponent exercise and mind–body exercise, is an effective prescription for improving the working memory of older adults (Cai et al. 2021).

### 1.4. Video Game Training

Video game training is another method of cognitive improvement that is well researched. In the last two decades, there has been an increasing interest in examining the effects of playing video games on cognition in the scientific community. Playing video games is popular among all ages, and around the world, much time is spent playing action video games; it is estimated that almost 3.2 billion people are video gamers worldwide (Video Game Industry Statistics 2022). Individuals’ ever-increasing use of video games has interested researchers in examining its cognitive effects. Action video games enhance the fundamental processes of retaining visual information in mind over a brief delay (Blacker et al. 2014). Action video games are fast-paced and require WM, such as keeping track of many items simultaneously, re-evaluating goals constantly and responding promptly to changing demands. For example, visual working memory is engaged when playing video games that requires users to store and retain task-relevant visual information for a period after that visual information has been removed. This ability to maintain visual information in working memory for a while is critical for learning new skills and solving novel problems (Alloway 2006; Logie 2014).

In the last decade, numerous meta-analytic studies have examined the effects of game training on cognition, and they have yielded mixed results. For example, in their meta-analysis, Powers et al. (2013) compared video game players with non-video game players in 71 quasi-experimental studies and reported a moderate to large effect size indicating that video game players have superior information-processing skills compared to non-video game players. Their second meta-analysis (Powers et al. 2013) examining the effects of video game training on 46 experimental studies found a small to medium effect size, indicating that video game training enhances information-processing skills. However, due to the inclusion of exergaming (games that require physical exercise as part of the gameplay) in their analysis, the actual effect size of the efficacy of video game training on cognitive functions is somewhat obscured.

In a study by Sala et al. (2018), three meta-analyses were conducted to explore the impact of video games on cognitive functions. The first meta-analysis included 66 correlational studies that assessed the correlation between video game skill and the cognitive ability of video game players. The studies involved 8141 cognitively and physically healthy participants, revealing a weak positive correlation between memory and video game skill and a significant correlation with spatial ability. However, from this analysis, it is not clear whether video game practice enhances spatial cognition or whether individuals with superior spatial skills are more likely to be skilled in playing video games. The second meta-analysis compared video game players with non-players in 98 quasi-experimental studies involving 6166 cognitively and physically healthy participants. The results showed that video game players outperformed non-players on memory and spatial ability measures. However, the third meta-analysis investigated the impact of video game training on the general cognitive ability of 63 studies, encompassing 3286 cognitively and physically healthy participants, and found near-zero training effects.

From the extent of the cognitive training literature so far, it is difficult to conclude whether certain types of training are effective in enhancing the WM functioning of physically and cognitively healthy adults. No study has compared different training methods from randomized controlled trials to improve the working memory (WM) performance of healthy individuals. Sala et al. (2019) conducted a second-order meta-analysis to evaluate the effectiveness of various working memory training programs in producing near- and far-transfer effects. However, their analysis included both clinical and non-clinical populations as well as children and older adults. Furthermore, as training methods have advanced over time, with the introduction of gamification in cognitive training and the study of approaches such as mindfulness training and physical activity training, there have been further meta-analyses (Gathercole et al. 2019; Cásedas et al. 2020; Yakobi et al. 2021) conducted since 2019 that Sala’s et al. (2019) second-order meta-analysis has not investigated.

Furthermore, their analysis did not specify randomized controlled trials in their inclusion criteria, and so the analyses included quasi-experimental studies. Randomized controlled trials are widely regarded as the gold standard in clinical research as they help reduce selection bias and confounding factors, leading to a more precise determination of causality (Chandler et al. 2019; Higgins et al. 2008). Consequently, an additional comprehensive second-order meta-analytic study is required to ascertain the true impact of working memory training programs on healthy individuals. The current second-order meta-analysis aims to evaluate and synthesize findings from meta-analyses of randomized controlled trials that investigated the efficacy of various training programs, such as mindfulness training, adaptive working memory training, physical activity training and video game training, in improving working memory capacity in physically and cognitively healthy individuals. It is hypothesized that, across studies, adaptive training with working memory tasks will show improvements in the working memory capacity of healthy adults, and to a greater extent than other types of training.

## 2. Method

### 2.1. Study Selection

An electronic database search for meta-analyses of randomized controlled trials examining the effects of interventions that aimed to improve working memory was undertaken in February 2022. The following electronic databases were searched: APA PsycINFO, ERIC and Medline. Keywords used for the study were “working memory”, “cognitive training”, “brain training”, “review”, and “meta-analysis”. These keywords were searched for the fields of title and abstract. Two authors (MS and JJ) independently completed the screening process. If the title and abstract of the study suggested that the paper may be appropriate for inclusion in the second-order meta-analysis, then the full-text was examined for inclusion criteria. The reference lists of identified studies were also checked for potentially relevant studies.

Meta-analyses were only included if they met the following criteria:

1. Randomized controlled trials (RCTs).
2. Publication in a peer-reviewed journal.
3. Participants must be healthy adults in the age range of 18 to 55 years.
4. Studies examining working memory task training, video game training, music intervention, exercise training, mindfulness training, physical activity training, commercially available WM training programs or any other training type that aims to improve working memory capacity were considered.

Our exclusion criteria were as follows:

1. Meta-analyses examining the effects of cognitive training on neural patterns.
2. Studies examining the effects of cognitive training programs on children (under 18 years) and older adults (over 55 years of age).
3. Studies examining the effects of cognitive training in individuals with developmental disabilities, neurological disorders, or progressive illnesses.
4. Studies examining the effects of interventions such as dietary supplements and pharmacological drugs.

### 2.2. Meta-Analytic Approach

From each study, we extracted the overall effect size and 95% confidence intervals summarizing the effects of the training programs on working memory between treatment and control groups. The effect size extracted from each meta-analysis was the standardized mean difference, computed as either Cohen’s d or Hedge’s g. Both effect sizes summarize the difference between groups in standard deviation units. We coded all effect sizes so that positive values indicated the training program was associated with superior working memory functioning compared to the comparison group. First, the effect sizes from each meta-analysis were averaged using a random effects model. Second, we also examined whether different interventions modulated effect sizes between meta-analyses. Following the approach by Tamim et al. (2011), we used the I^2^ statistic for this calculation. In first-order meta-analyses, the I^2^ statistic measures the percentage of heterogeneity arising from systematic influences. For instance, an I^2^ value of 100 indicates that all the differences between study-level effect sizes are due to systematic factors, while an I^2^ value of zero suggests that these differences are solely due to sampling error. By applying this statistic to the average effect sizes from the first-order meta-analyses, we can determine the percentage of heterogeneity between meta-analyses resulting from between-meta-analysis errors or systematic influences. For example, an I^2^ value of zero would indicate that variability among first-order meta-analyses can be attributed to second-order sampling error. In this report, if the I^2^ value were not significantly different from zero, it would imply that the type of working memory intervention/training program moderated working memory performance. All analyses used Comprehensive Meta-Analysis (Borenstein et al. 2022).

**Figure 1:**
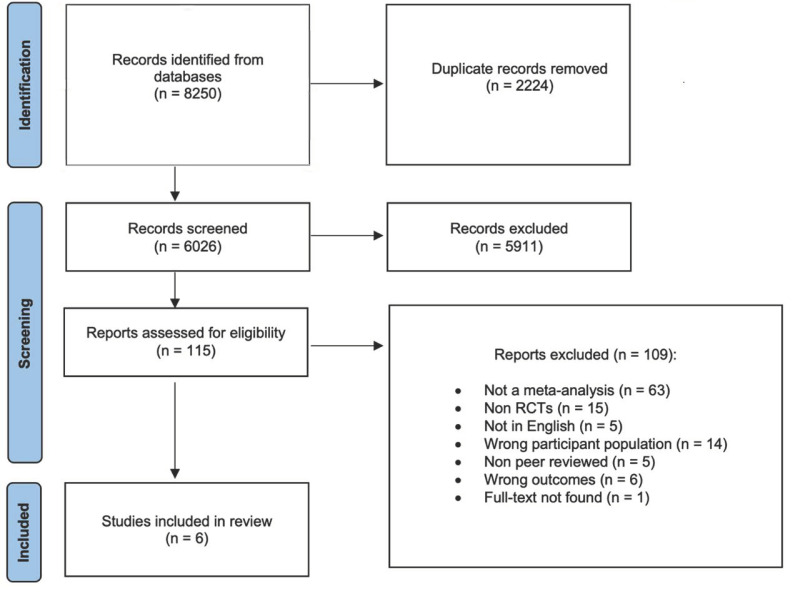
PRISMA flowchart showing process of identifying articles for the second-order meta-analysis.

Moreover, interpreting effect sizes, according to Ferguson (2016), helps us better understand the magnitude of differences between various groups in experimental studies. An effect size of 0.41 is deemed small but still holds practical significance (Ferguson 2016). An AMSTAR 2 assessment (Shea et al. 2017) evaluated the included studies and determined that the studies by Wilke et al. (2019), Vermeir et al. (2020), and Cásedas et al. (2020) were of high quality and methodologically sound, with transparent methods. The studies by Yakobi et al. (2021), Soveri et al. (2017) and Gathercole et al. (2019) were categorized as moderate quality due to missing critical steps in their meta-analysis, such as incomplete data extraction by two independent authors. The AMSTRAR-2 assessment is included as a supplemental file. Figure 1 presents a PRISMA flowchart that outlines the process for identifying articles for the second-order meta-analysis.

## 3. Result

A total of 8250 records were found through a database search, and after screening, six studies were included in the final sample for analysis. Three studies included healthy and clinical samples (Gathercole et al. 2019; Cásedas et al. 2020; Vermeir et al. 2020). We included these meta-analytic studies in our analysis because most of their included individual studies had healthy samples. Three of the included studies had participants less than 18 years of age (Soveri et al. 2017; Vermeir et al. 2020; Gathercole et al. 2019), and all studies had participants who were older than 55 years of age. However, most participants in the included studies fell within the age range of 18 and 55 years. Studies where we noticed that the majority of the individual studies had healthy participants in the age range between 18 and 55 years were included in our analysis, while a minority of studies had participants who had clinical presentation and were over the age of 55 years or less than 18 years of age. We identified two studies examining the efficacy of mindfulness training on working memory: two studies utilized adaptive working memory training, one study utilized resistance training (single bout), and one study utilized computerized cognitive training (gamification) as an intervention (See Table 1 for a summary of study characteristics). In Table 1, the total number of samples (n) and the total number of included studies (k) in individual meta-analyses are accurately reported. However, the total sample size for the Soveri et al. (2017) study was unavailable, so we calculated it by reviewing the individual studies. We found that only 25 of the included studies had working memory as an outcome variable. Among these, there were 3 unpublished studies that we were unable to locate. Therefore, the total sample size of 1430 is based on 23 studies, excluding the samples from these three unpublished studies. Two of the meta-analyses included in this study evaluated the impact of adaptive cognitive training on working memory tasks (Soveri et al. 2017; Gathercole et al. 2019). Gathercole et al. (2019) assessed various working memory tasks including the n-back task, complex span task, running span task, updating tasks, Cogmed QM, and Cogmed RM. Meanwhile, Soveri et al. (2017) focused specifically on the n-back working memory task.

The overall second-order average effect size was found to be small yet statistically significant (SMD = 0.335, 95% CI [0.223; 0.447], *p* < .001). Significant levels of heterogeneity were also found. The I^2^ statistic was 67.9% and significant (Q = 18.687, *p* = .005). Pairwise comparisons of effect sizes revealed that, out of the four trainings examined, working memory task training is significantly different from controls (SMD = 0.370, SE = 0.07, 95% CI [0.223; 0.507], *p* < .001) and is effective in improving working memory performance. Results show no significant difference in working memory outcomes between the mindfulness training group and the control group following mindfulness training (SMD = 0.248, SE = 0.131, 95% CI [−0.009; 0.506], *p* = 0.058). Moreover, no significant difference was observed for resistance exercise training (SMD = 0.350, SE = 0.204, 95% CI [−0.05; 0.750], *p* = 0.086) and gamification (SMD = 0.210, SE = 0.224, 95% CI [−0.230; 0.650], *p* = 0.350).

The next set of analyses comprised head-to-head tests to determine superiority effects with respect to each of the studied interventions. The results of these tests are summarized in Table 2. These analyses showed no significant differences between any pairs of comparisons.

## 4. Discussion

A substantial body of the literature has investigated the impact of working memory training programs on different outcome variables across diverse age groups and populations. The studies have aimed to elucidate the efficacy of different training methods in improving working memory performance (Cásedas et al. 2020; Soveri et al. 2017; Schwaighofer et al. 2015). The objective of the current second-order meta-analysis was to assess the efficacy and superiority of different training programs, namely mindfulness training, computerized working memory adaptive training, physical activity training, and video game training, in improving the working memory capacity of healthy adults. Our literature review identified six meta-analyses of randomized controlled trials on working memory outcomes from different types of training.

We found a small but significant positive effect, showing limited working memory performance improvement following training. Further examining individual interventions, working memory training has the most robust and reliable effect compared to control conditions. This finding is consistent with previous second-order meta-analytic investigations that reported a small near-transfer effect on working memory outcomes (Sala et al. 2019). Next, we completed head-to-head tests to examine which training is superior in improving working memory capacity, but our small number of meta-analyses limited our sensitivity to potential differences between intervention types. We observed no significant differences in effectiveness between the interventions, but we did not have the power and sensitivity to detect the difference between different interventions at this stage.

Numerous previous studies have aimed to improve working memory capacity through training. Multiple studies suggest a small, measurable, positive effect of training on WM capacity, and research has found that working memory training produces a near-transfer effect, a transfer effect on a task similar to the training task (Melby-Lervåg and Hulme 2013; Sala et al. 2019; Soveri et al. 2017). In a second-order meta-analysis, Sala et al. (2019) synthesized findings from individual meta-analyses of controlled trials. They found a small near-transfer effect size of g = 0.20 for adults. Our research revealed a slightly larger effect size of SMD = 0.335. There are several potential reasons for this minor variance in effect sizes. For example, our study exclusively focused on randomized controlled trials, whereas Sala et al. (2019) included quasi-experimental studies. Randomization of participants enhances validity, reduces the risk of selection bias, and helps account for confounding variables, so our findings may be more reliable despite having less power. Additionally, our research considered more recent studies that may have yielded improved training effects due to improved training approaches.

Our study did not examine the issue of transfer from training tasks. However, in their second-order meta-analysis, Sala et al. (2019) reported an absence of a far-transfer effect, indicating a lack of transfer effect on tasks dissimilar to the training task, such as inhibitory control tasks and fluid intelligence. The authors argue that the presence of a near-transfer effect and an absence of a far-transfer effect suggests that while human cognition is trainable, the benefits of training are mainly specific to the trained domain. Similarly, in a meta-analysis, Soveri et al. (2017) discovered small transfer effects of working memory training to untrained working memory tasks, fluid intelligence tasks, and cognitive control tasks in healthy adults. Their conclusion suggests that the training may have general effects, such as improved attention and perceptual speed, which resulted in minimal improvement on measures unrelated to the training task, such as fluid intelligence and cognitive control tasks. However, they noted that these minor improvements in other cognitive measures may have limited practical significance.

However, the research continues to suggest possibilities. A meta-analysis by Gathercole et al. (2019) investigated how different working memory training tasks, including the input modality (auditory or visual), recall modality (spoken), type of stimulus used in training (words or numbers), and the format in which the stimulus is presented (verbal or visuospatial), may predict the extent to which transfer occurs. The analysis focused on working memory training programs within RCTs, incorporating simple/complex span, running span, n-back, and Cogmed training programs and found a small to moderate near-transfer effect to both trained and untrained working memory tasks (d = 0.42). Gathercole and colleagues suggested that the transfer of skills was significant for complex span tasks when both the trained and untrained tasks used material in the same domain, such as verbal or visuospatial. The authors also noted that training on complex span tasks, which led to developing new mental routines, resulted in significant improvement. For example, a greater transfer effect was observed in the backward span task, as the task demands more effort and strategic thinking for the trainee to recall the list of items backward. This suggests that tasks requiring more cognitive effort lead to the development of new routines, predicting a larger transfer effect. Further, in the serial recall task, it was observed that there was a considerable transfer of learning for spatial material and, to a somewhat lesser extent but still substantial, for verbal material. This phenomenon is attributed to the fact that verbal material taps into existing cognitive routines, as we tend to rely more on verbal information in our daily lives, whereas visuospatial information may encourage trainees to develop new strategies during training.

Still, other studies provide some optimism for elusive far-transfer. Although far-transfer effects on general cognitive abilities such as fluid intelligence and inhibitory control tasks have not been observed following training, there have been reports of far-transfer effects to other aspects of daily functioning and indices of wellbeing (Spencer-Smith and Klingberg 2015; Barkus 2020; Takeuchi et al. 2014; Whitelock et al. 2018) suggesting that an enhancement in working memory capacity, or evidence of a near-transfer effect, itself may be significant for the trained individual. For instance, Spencer-Smith and Klingberg (2015) in a meta-analysis of twelve randomized controlled studies, discovered that working memory training has wide-ranging benefits, leading to improvements in daily functioning by reducing inattention in daily life in both healthy individuals and clinical populations across all age groups, including children, adolescents, and adults. Spencer-Smith and Klingberg (2015) observed that the near-transfer effect to both visuospatial and verbal working memory led to generalized improvements in reducing casual inattention, suggesting that the benefits of working memory training extend to everyday functioning. Furthermore, the authors reported long-term maintenance of transfer effects that persisted at 2–8 months follow-up.

Moreover, research indicates that working memory training may have wide-ranging benefits, including improvements in mood (Cui et al. 2024; Pan et al. 2022; Venter 2021; Barkus 2020; Xiu et al. 2016; Diamond et al. 2015; Takeuchi et al. 2014). Specifically, training that focuses on updating working memory capacity positively impacts mood. Updating working memory training involves practicing updating information in working memory, for example, through tasks like adaptive n-back training or letter memory tasks (Xiu et al. 2016). This improved ability to update information in working memory can help disengage from negative emotional events more quickly and successfully update emotional information, which aids in breaking the cycle of rumination (Xiu et al. 2016; Wang et al. 2019). The positive impact of working memory training on mood has been connected to increased efficiency of the frontoparietal cognitive control network, as evidenced by fMRI scans, leading to improved capacity for emotion regulation among the trained individuals (Schweizer et al. 2013). Working memory training has been linked to changes in emotion regulation strategies, resulting in enhanced efficiency in approaching and handling emotional material among participants who received the training (Barkus 2020). The underlying mechanism suggests that working memory and emotion regulation share a common neural substrate when handling emotional stimuli. Consequently, training in one (WM or emotional regulation) can benefit the other by enhancing the efficiency of this shared neural network (Schweizer et al. 2013).

All in all, the findings of the current second-order meta-analysis suggests that working memory capacity of healthy adults can be improved through training. However, the effect size observed was small indicating that the practical improvements for individuals in real-life settings may be limited. Four different interventions that aim to improve working memory capacity were compared, including adaptive cognitive training with WM tasks, physical activity training, mindfulness training and video game training. At this stage, no single type of training was found to be superior to the others.

## 5. Limitations

We observed significant heterogeneity among the studies included in our second-order meta-analysis, suggesting considerable variability in effect sizes across different studies. Previous meta-analyses have indicated that age can serve as a crucial moderator of working memory training success, with younger children demonstrating greater improvements in working memory tasks following training compared to adults (Melby-Lervåg and Hulme 2013). Additionally, the design of the study plays an important role; studies utilizing passive control groups generally report larger effects than those employing active control groups (Melby-Lervåg et al. 2016). Our study incorporated a blend of active and passive control conditions, primarily targeting healthy adults aged 18 to 55. However, it also encompassed some meta-analyses that examined training effects on the younger population. For instance, meta-analyses by Soveri et al. (2017), Vermeir et al. (2020), and Gathercole et al. (2019) comprised samples of children and young adults under 18. The inclusion of older adults over 55 years across several meta-analyses further contributed to the observed heterogeneity (Sala et al. 2019). At this point, we were unable to conduct a moderator analysis to identify the underlying sources of this heterogeneity, as we included only six meta-analyses in our second-order meta-analysis. According to Cochrane Collaboration guidelines (Cumpston et al. 2022), a minimum of ten studies is required to execute a moderator analysis, leading us to refrain from performing one in this instance.

Furthermore, some studies included in our analysis reported multiple effect sizes. Our findings were based on the overall effect sizes provided by each meta-analysis, which employed a random effects inverse variance model. However, it remains unclear how the individual studies addressed nested effect sizes when consolidating their results, as this information was not consistently reported across the meta-analyses we reviewed. We utilized the Comprehensive Meta-Analysis (CMA) software to manage nested effect sizes, but this software does not accommodate more advanced methods like Robust Variance Estimation (RVE). Consequently, this limitation could introduce additional variability into the overall effect size estimates.

## Figures and Tables

**Table 1:** Characteristics of included studies.

Study	Intervention Type	Comparison Condition	n	k	Age	Clinical/Healthy	Outcome	Average Effect Size (SMD)	Standard Error
Gathercole et al. (2019)	Working memory training	Active only	1406	23	4–77 years	Both	Working memory task	0.37	0.07
Cásedas et al. (2020)	Mindfulness Training	Active/passive	1112	4	20.3–73.4 years	Both	Working memory task	0.42	0.163
Yakobi et al. (2021)	Mindfulness-Based Intervention	Active/passive	1632	8	18–65 years	Healthy	Working memory task	0.148	0.085
Wilke et al. (2019)	Resistance Exercise (single bout)	Crossover/Parallel-group	465	3	20.4–72.3 years	Healthy	Working memory task	0.35	0.204
Vermeir et al. (2020)	Computerized Cognitive Training (Gamification)	Active	231	4	8.98–82.7 years	Both	Working memory task	0.21	0.225
Soveri et al. (2017)	Working memory training	Active/passive	1430	25	59 years and younger, 60 years and over	Healthy	Working memory task	0.24; 0.62	0.041, 0.094

**Table 2:** *p*-values for the head to head comparison.

	Working Memory Training	Mindfulness Training	Resistance Exercise (Single Bout)
Working Memory Training	-		
Mindfulness Training	0.413	-	
Resistance Exercise (single bout)	0.926	0.675	-
Gamification	0.496	0.883	0.644

## Data Availability

Not applicable.

## Supplementary Materials

The following are available online at https://www.mdpi.com/article/10.3390/jintelligence12110114/s1, Table S1. The AMSTRAR-2 assessment.
